# Alterations in Gene Expression of Renin-Angiotensin System Components and Related Proteins in Colorectal Cancer

**DOI:** 10.1155/2021/9987115

**Published:** 2021-07-05

**Authors:** Danial Mehranfard, Gabriela Perez, Andres Rodriguez, Julia M. Ladna, Christopher T. Neagra, Benjamin Goldstein, Timothy Carroll, Alice Tran, Malav Trivedi, Robert C. Speth

**Affiliations:** ^1^College of Pharmacy, Nova Southeastern University, Fort Lauderdale, FL, USA; ^2^Department of Internal Medicine, Palmetto General Hospital, Hialeah, FL, USA; ^3^Department of Internal Medicine, University of Miami/Jackson Memorial Hospital, Miami, FL, USA; ^4^Broward Health Medical Center, Fort Lauderdale, FL, USA; ^5^Baylor College of Medicine, Houston, TX, USA; ^6^University of Florida, Gainesville, FL, USA; ^7^College of Psychology, Nova Southeastern University, Fort Lauderdale, FL, USA; ^8^Halmos College of Arts and Sciences, Nova Southeastern University, Fort Lauderdale, FL, USA

## Abstract

**Materials and Methods:**

Quantitative expression of the RNA of these 17 genes in normal and cancerous tissues obtained using chip arrays from the public functional genomics data repository, Gene Expression Omnibus (GEO) application, was compared statistically.

**Results:**

Expression of four genes, *AGT* (angiotensinogen), *ENPEP* (aminopeptidase A) *MME* (neprilysin), and *PREP* (prolyl endopeptidase), was significantly upregulated in CRC specimens. Expression of *REN* (renin), *THOP* (thimet oligopeptidase), *NLN* (neurolysin), *PRCP* (prolyl carboxypeptidase), *ANPEP* (aminopeptidase N), and *MAS1* (Mas receptor) was downregulated in CRC specimens.

**Conclusions:**

Presuming gene expression parallel protein expression, these results suggest that increased production of the angiotensinogen precursor of angiotensin (ANG) peptides, with the reduction of the enzymes that metabolize it to ANG II, can lead to accumulation of angiotensinogen in CRC tissues. Downregulation of *THOP*, *NLN*, *PRCP*, and *MAS1* gene expression, whose proteins contribute to the ACE2/ANG 1-7/Mas axis, suggests that reduced activity of this RAS branch could be permissive for oncogenicity. Components of the RAS may be potential therapeutic targets for treatment of CRC.

## 1. Introduction

### 1.1. Colorectal Cancer

Colorectal cancer is the second leading cause of cancer-related deaths in the USA and it is the third most common cancer in males and in females [1]. Globally, it is the second leading cause of cancer in females and third leading cause in males, with over half of the cases occurring in developed regions [2]. While there has been a large focus on CRC prevention by screening modalities, much remains undiscovered regarding better treatment options for this often-fatal disease. The current gold standard modality for diagnostic screening and early intervention is colonoscopy. With colonoscopies, physicians can directly visualize, locate, biopsy, and resect areas of concern. The incidence for CRC has decreased by 6.24% between 2005 and 2017 in both genders, across all ages and ethnicities [3]. However, the prevalence of the disease remains high. Due to socioeconomic factors such as barriers to initial screening and access to follow-up care, CRC contributes to a lethal diagnosis in ~20% of newly diagnosed colon cancers, as many have already metastasized at initial presentation [4]. The use of lower-cost screening methods, such as fecal immunochemical tests (FIT) [5] or epigenetic changes and fecal hemoglobin, e.g., Cologuard®, while not as accurate as colonoscopy, is an option that is FDA approved [6]. Many CRC patients also have genetic predispositions and increased lifestyle risk factors for CRC including alcohol and tobacco use, lack of physical activity, and obesity [4]. Current treatment options vary depending on the stage of the disease. CRC lesions are staged using TNM (primary tumor (T), regional lymph node involvement (N), and distant metastasis (M)) staging of the combined American Joint Committee on Cancer (AJCC)/Union for International Cancer Control (UICC) [7]. According to the National Comprehensive Cancer Network guidelines (http://NCCN.org), treatment will depend on the stage and location of the disease as well as in-patient factors. Generally, for localized colon cancer, the curative treatment is surgical resection in surgical candidates. Nonsurgical options are available for patients with more advanced cancer. For patients with cancer that has metastasized, the therapy is surgery and/or adjunctive chemotherapy and radiation, depending on the stage of the disease. The current treatment options are invasive, and patients experience adverse side effects such as pain, disruption of their alimentary system, the need for colostomy bags, and systemic side effects of chemotherapy and radiation. Our study findings imply that already-existing, noninvasive, and well-tolerated therapies may be of benefit for the prevention and adjunctive treatment of CRC.

#### 1.1.1. Pathophysiology of Colorectal Cancer

As reviewed by Cappell [8], the pathophysiology of CRC is well established. It can arise from a variety of mechanisms including sporadic mutation and familial syndromes or originate from a serrated hyperplastic polyp or adenomatous polyp (AP) through the adenoma-carcinoma sequence. The National Cancer Institute's (NCI's) Physician's Data Query (PDQ) cancer information summary about CRC states that the majority of colon cancers today arise from an AP through the adenoma to carcinoma sequence, although serrated-type hyperplastic polyps can also transform into CRC via a BRAF (B-Raf proto-oncogene serine/threonine kinase) mutation [9]. Molecular transformations such as those seen in epigenetic alterations, e.g., DNA methylation defects and microRNA instability, which can be affected by lifestyle and environmental factors, play a role in the development and pathogenesis of CRC as well [10]. Physicians routinely screen for the presence of APs with colonoscopy and the lesions are easily biopsied during the procedure for histological evaluation. Histological evaluation is imperative to determine the malignant potential of the cells [8]. Once a lesion is biopsied and evaluated histologically, the sample is classified. Adenomatous polyps can be classified as tubular, villous, or tubulovillous, with the villous subtype having a high risk for transformation to cancer [11].

Syndromes that place patients at high risk for CRC include familial adenomatous polyposis (FAP), the result of an autosomal dominant (AD) germline mutation of the APC (adenomatous polyposis coli) gene on chromosome 5q, and hereditary nonpolyposis colon cancer (HPNCC) that is a result of mutated mismatch repair genes; see reviews [12, 13]. Patients with FAP will inevitably develop CRC via the growth of hundreds of colonic adenomas after puberty, and they require colectomy at a young age to prevent the inevitable development of CRC. Patients with HNPCC will not have the growth of hundreds of polyps typically seen in FAP patients; however, they will have growth of several, usually right-sided, sessile polyps during their middle-aged years. This, with the use of Amsterdam II diagnostic criteria [8], enables practitioners to establish a clinical diagnosis of HNPCC.

Syndromic CRCs have allowed for the study and understanding of sporadic CRCs. We now understand that colon cancer is the result of a cascade of mutations that eventually lead to accelerated colonic cell multiplication, such as the mutations that occur in familial syndromes. In sporadic CRC, mutations of genes including the APC regulatory gene, k-ras cell-signaling gene, P53 or DCC tumor suppressor genes, or the mutation of mismatch repair genes may spontaneously occur and lead to CRC in patients without a germline mutation. Environmental factors also play a role in the evolution of sporadic CRC via the DNA methylation process that can lead to the inactivation of tumor suppressor genes by hypermethylation of the CpG islands in their promoter regions [14].

### 1.2. Renin-Angiotensin System

**Figure 1:**
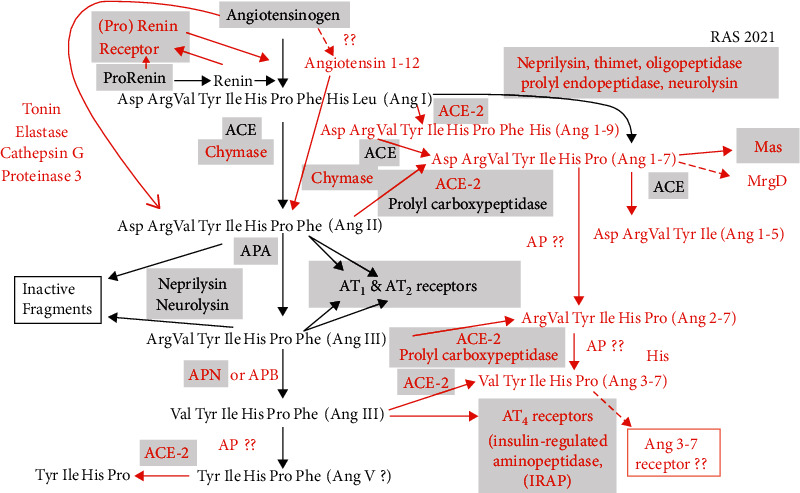
Current understanding of the renin-angiotensin system 2021. Black font indicates the classical RAS circa 1990. Red font indicates additional metabolic and signaling pathways that have been incorporated into the RAS since 1990. Proteins, enzymes, and receptors indicated in gray boxes are the proteins whose genes were evaluated in this analysis. Dotted lines indicate hypothetical pathways. ^??^Uncharacterized enzymatic mediators.

Several studies describe the possible involvement of the RAS in the pathophysiology of CRC [15–18]. While the RAS is primarily associated with the regulation of the cardiovascular system as well as fluid and electrolyte balance, we now know it to be involved in a wide range of cellular processes [19–21]. The modern-day RAS as shown in Figure 1 is a complexly organized system with many divergent pathways. Indeed, some of the enzymatic pathways of the RAS intersect with other metabolic pathways, e.g., ACE is also kininase II which metabolizes bradykinin and several other peptides; neprilysin, which forms Ang 1-7 from Ang I, also degrades atrial natriuretic peptide and several other peptides; neurolysin, which metabolizes Ang I to Ang 1-7 and Ang II at the Tyr-Ile bond, metabolizes neurotensin and other peptides. The signaling pathways of the RAS through its receptors, AT_1_, AT_2_, AT_4_, Mas, and MrgD, are also complex and divergent, involving G protein-activated enzymatic pathways, beta-arrestin-activated pathways, transactivation of EGF, and activation of NADPH oxidase, as reviewed [22–25]. Dysregulation of the RAS has been implicated in several cancers, including CRC, lung cancer, and gliomas [26–29].

### 1.3. Constituents of the “Classical” and “Neoclassical” Renin-Angiotensin System

#### 1.3.1. Angiotensinogen (AGT) and Renin (Classical): *AGT* and *REN*

Angiotensinogen is an essential component of the RAS. It is the sole precursor of all of the angiotensin peptides, which play a critical role in the regulation of blood pressure as well as fluid and electrolyte balance, primarily through the actions of angiotensin II (Ang II) acting on the AT_1_ Ang II receptor subtype (AT1R) [30]. Human AGT is primarily, but not exclusively, synthesized in the liver and constitutively secreted into the bloodstream. It has 485 amino acids, including a 33-amino acid signal peptide that is cleaved prior to circulation in the bloodstream. Circulating AGT is cleaved by renin to produce angiotensin I (Ang I), a 10-amino acid peptide cleaved from the N terminus of this protein. Renin is an aspartyl protease that is synthesized predominantly in the juxtaglomerular apparatus of the kidney and secreted into the circulation as the mature enzyme. The precursor protein (prorenin) is secreted from several tissues other than the juxtaglomerular apparatus including the adrenal gland, ovary, testis, placenta, retina, brain [31, 32], and collecting duct [33]. Renin is responsible for the cleavage of angiotensinogen to form Ang I. Prorenin (see also the next section), when bound to the prorenin receptor prorenin, can also form Ang I from angiotensinogen [34].

#### 1.3.2. Prorenin Receptor (Neoclassical): *ATP6AP2*

Prorenin is the precursor of renin. It exists in circulating blood at concentrations that are 5 to 10 times higher than those of renin [35]. Previously, prorenin was considered to be an inactive form of renin with no physiological role. However, it has been known for some time that circulating levels of prorenin are elevated in diabetic subjects [36]. We now know that the prorenin receptor binds prorenin, uncovering the active site of the enzyme, as well as renin, subsequently enabling prorenin to cleave Ang I from angiotensinogen [34]. When prorenin and renin bind to the prorenin receptor, they also activate a protein kinase cascade response [34]. In addition to activating prorenin and generating an intracellular response [34], the prorenin receptor is also an accessory protein component of the V-ATPase proton pump ATPase 6 and a component of the Wnt signaling pathways; see review [37].

#### 1.3.3. Angiotensin-Converting Enzyme (ACE) (Classical): *ACE*

Ang I is converted to Ang II, the primary hormone of the RAS, predominantly by ACE, a di-peptidyl carboxypeptidase also known as kininase II based upon its ability to metabolize bradykinin. It also metabolizes other peptides, notably substance P and enkephalins [38].

#### 1.3.4. Chymase (Neoclassical): *CMA1*

Chymase is a serine protease that cleaves Ang I at the same site as ACE. Due to a high affinity for angiotensin I, chymase converts Ang I to Ang II at a substantially greater rate than does ACE [39]. Chymase is expressed in mast cells and is thought to also function in the degradation of the extracellular matrix and the regulation of submucosal gland secretion, as well as to oppose inflammation by inactivating allergens and neuropeptides causing inflammation [40]. While chymase has yet to be implicated in any cancers, it has been shown that chymase expression is upregulated in the human diabetic kidney, specifically in mesangial cells and vascular smooth muscle cells [41, 42] and in polycystic kidney disease [41].

#### 1.3.5. Angiotensin-Converting Enzyme-2 (ACE2) (Neoclassical): *ACE2*

ACE2, while having a high homology to ACE, is a monocarboxypeptidase and has an entirely different spectrum of activity. It is primarily known for its ability to convert Ang II to angiotensin 1-7; although, it can also convert Ang I to angiotensin 1-9 [43, 44]. While unrelated to its functionality in the RAS, ACE2 is also notable for its role as the primary receptor for SARS and SARS-CoV-2 coronaviruses [45, 46].

#### 1.3.6. Prolylcarboxypeptidase (Angiotensinase C) (Classical): *PRCP*

Prolylcarboxypeptidase is one of several enzymes capable of cleaving a post proline amino acid. It was previously known as angiotensinase C based upon its ability to efficiently cleave the carboxy-terminal phenylalanine from Ang II [47].

#### 1.3.7. AT_1_ Receptor, Ang II Receptor Subtype (Classical): *AGTR1*

The AT_1_ receptor is the primary mediator of Ang II. It causes vasoconstriction, sodium retention, thirst, salt appetite, and aldosterone synthesis and release. It has a variety of signaling pathways including mobilization of intracellular calcium, opening of calcium ion channels, activation of NADPH oxidase, transactivation of the EGF receptor, and activation of mitogen-activated protein (MAP) kinase cascades [48]. Increased activation of EGF receptors and mitogen-activated protein kinases is found in many cancer cell types [49]. Additionally, the AT_1_ receptor has some constitutive activity as well as being activated by stretch independently of Ang II [50].

#### 1.3.8. AT_2_ Receptor, Ang II Receptor Subtype (Classical): *AGTR2*

The AT_2_ receptor also has a G protein-coupled receptor motif, but it behaves in an idiosyncratic fashion [51, 52]. The most interesting characteristic of the AT_2_ receptor is that its actions tend to oppose those of the AT1R [50, 53]. For example, the AT_2_ receptor activates phosphatase activity and opens a potassium channel, which inhibits cellular activation [54]. The AT_2_ receptor is highly expressed in utero [55, 56], but it has a limited expression in the postnatal period. It is also expressed on atretic follicles [57]. Like the AT_1_ receptor, the AT_2_ receptor is also constitutively active and may respond to ligands other than Ang II [50, 58].

#### 1.3.9. MAS Protein (Ang 1-7 Receptor) (Neoclassical): *MAS1*

Mas encodes a class A seven-transmembrane-spanning G-protein-coupled receptor, identified as a receptor for Ang 1-7 [59], which is a peptide derived from Ang II by the actions of ACE2 [43] and prolylcarboxypeptidase, formerly known as angiotensinase C [47]. Mas plays a role in multiple processes, including vasodilation with reduction of blood pressure, thereby exhibiting cardioprotective properties by mediating the effects of Ang 1-7 [59]. Thus far, a decrease in MAS1 expression has been associated with tumor growth, lymph node metastasis, and grade of invasive ductal carcinoma [60], while treatment with Ang 1-7 is reported to reduce breast tumor volume [61].

#### 1.3.10. Aminopeptidases (Neoclassical)

The two primary aminopeptidases acting upon angiotensin peptides are aminopeptidase A (APA), encoded by *ENPEP*, and aminopeptidase N (APN), encoded by *ANPEP*.

Aminopeptidase A, also known as glutamyl aminopeptidase, releases amino-terminal Glu and Asp residues from proteins and peptides. Aminopeptidase A is found diffusely throughout the brush borders of intestinal enterocytes [62]. Aminopeptidase A converts Ang II to angiotensin III (Ang III), which is reported to be equipotent to Ang II at both the AT_1_ and AT_2_ receptors [63]. In the brain, Ang III is reported to be the primary effector of vasopressin release [64, 65] although this has been disputed [66].

Aminopeptidase N, also known as CD13, is a multifunctional enzyme that is present in many different human tissues. It serves as a receptor for several viruses including a coronavirus that causes colds [67, 68]. It plays a significant role in trimming of antigens and is involved in antigen presentation; it can also influence immune functions including angiogenesis and cell proliferation [67]. Aminopeptidase N is known to serve a role in the processing of various peptides including conversion of Ang III to angiotensin IV (Ang IV) as well as metabolizing different chemokines and playing a role in the final digestion of peptides derived from gastric and pancreatic processes [69]. With respect to the RAS, its primary role is to metabolize Ang III to Ang IV, which terminates the ability of the Ang peptide to activate the AT_1_ and AT_2_ receptors. Metabolism of Ang IV to the pentapeptide (Ang 4-8) and smaller fragments by other aminopeptidases generates angiotensin peptides for which no function has yet been identified.

#### 1.3.11. AT_4_ Receptor, Insulin-Regulated Aminopeptidase (Neoclassical): *LNPEP*

The AT_4_ receptor, akin to the prorenin receptor, was previously characterized under a different name based upon a different functionality. The AT_4_ receptor is better known as insulin-regulated aminopeptidase (IRAP) [70, 71]. It is a membrane-bound aminopeptidase that associates with GLUT-4, which is involved in glucose transport. It is a multifunctional peptidase whose substrates include vasopressin and oxytocin. When Ang IV binds to IRAP, it inhibits its peptidase activity. It is suggested that the pharmacological actions of Ang IV may be attributable to an increased abundance of IRAP's substrates [72]. A second type of receptor for Ang IV was identified as c-met, the receptor for the hepatocyte growth factor [73], at which Ang IV is also reported to act as an inhibitor. Additional potential receptors for Ang IV are several Mas-related G protein-coupled receptor-like proteins, e.g., MrgD, MrgH, and MRG [74].

#### 1.3.12. Endopeptidases That Act upon Angiotensin Peptides

Several endopeptidases metabolize angiotensin peptides. With respect to the functionality of the RAS, four endopeptidases metabolize Ang I to Ang 1-7: neprilysin, thimet oligopeptidase, neurolysin, and prolyl endopeptidase [75].

*(1) Neprilysin (Neoclassical)*. *MME* is a neutral endopeptidase that is highly expressed in kidney and lung tissues. Neprilysin is responsible for inactivating many regulatory peptides of the mammalian nervous, cardiovascular, inflammatory, and immune systems [76]. By inhibiting neprilysin, the bioavailability of natriuretic peptides, bradykinin, and substance P increases. As a result, these effects allow an effective antihypertensive response. A neprilysin inhibitor (sacubitril) is being used clinically to treat congestive heart failure in combination with the angiotensin receptor blocker valsartan [77]. The beneficial effects of neprilysin inhibition suggest that the preservation of natriuretic peptides outweighs the reduction in Ang 1-7 formation from Ang I.

*(2) Thimet Oligopeptidase (Neoclassical)*. *THOP* is a neuropeptidase in the metallopeptidase family that is responsible for forming enkephalins, while degrading other peptides [78]. Thimet oligopeptidase preferentially metabolizes neuropeptides under 20-amino acid residue long and forms Ang 1-7 from Ang I [79].

*(3) Neurolysin (Neoclassical)*. *NLN* is an oligopeptidase that hydrolyzes many different peptides including neurotensin, bradykinin, and dynorphin A [80]. Neurotensin is particularly important because it regulates luteinizing hormone (LH), prolactin release, and blood pressure; it may also be neuroprotective in stroke [81]. It can both form Ang 1-7 from Ang I as well as cleave Ang II and likely other angiotensin peptides at the Tyr-Ile bond [80].

*(4) Prolyl Endopeptidase (Prolyl Oligopeptidase) (Neoclassical)*. *PREP* is serine peptidase that cleaves peptides distal to the carboxy end of a proline [82]. It can metabolize both Ang I and Ang II to form Ang 1-9 and Ang 1-7. It also can metabolize Ang III and Ang IV to the corresponding des Phe metabolites [82].

## 2. Materials and Methods

**Figure 2:**
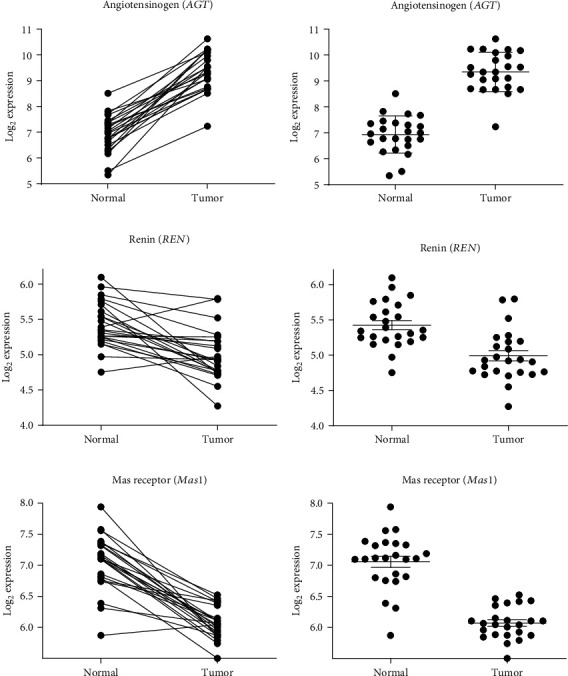
RAS components that showed highly significant differences in gene expression between normal and tumor tissues. (a, b) Describe angiotensinogen gene (*AGT*) expression, (c, d) describe renin gene (*REN*) expression, and (e, f) describe Mas receptor gene (*MAS1*) expression. (a, c, and e) Show pairing of samples with connecting lines. (b, d, and f) Show mean, SEM, and individual data points. All of these comparisons were significant at the *p* < 0.01 level after correction for multiple comparisons.

**Figure 3:**
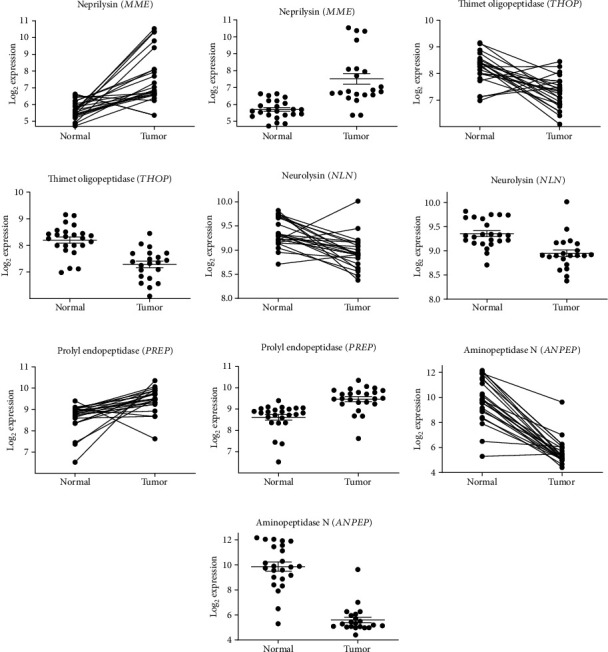
RAS-related enzymes that showed highly significant differences in gene expression between normal and tumor tissues. (a–j) Describe neprilysin gene (*MME*) expression, thimet oligopeptidase gene (*THOP*) expression, neurolysin gene (*NLN*) expression, prolyl endopeptidase gene (*PREP*) expression, and aminopeptidase N gene (*ANPEP*) expression. (a, c, e, g, and i) Show pairing of samples with connecting lines. (b, d, f, h, and j) Show mean, SEM, and individual data points. All of these comparisons were significant at the *p* < 0.01 level after correction for multiple comparisons.

**Figure 4:**
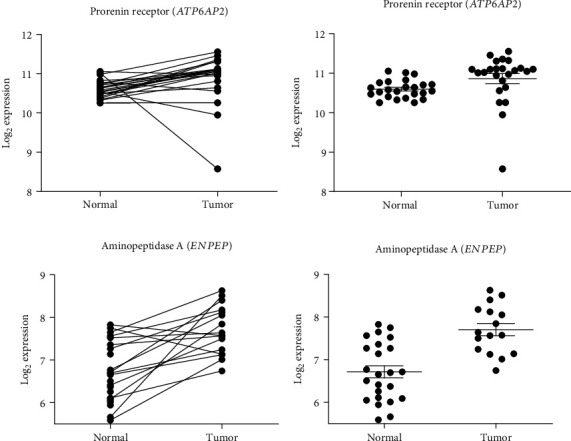
RAS-related components that showed significant (*p* < 0.05) differences in gene expression between normal and tumor tissues. (a, b) Describe prorenin receptor gene (*ATP6AP2*) expression, and (c, d) describe aminopeptidase A gene (*ENPEP*) expression. (a, c) Show pairing of samples with connecting lines. (b, d) Show mean, SEM, and individual data points. All of these comparisons wesssre significant at the *p* < 0.05 level after correction for multiple comparisons.

Seventeen genes (Figures 2–4, S1-S2) of the RAS and related enzymes were selected for analysis in 148 laser capture microresected (LCM) and homogenized tissue samples of male patients with CRC [83]. The quantitative expression of the RNA of these 17 genes in normal and cancerous tissue samples was obtained using chip arrays from the public functional genomics data repository, Gene Expression Omnibus (GEO) application. There were 24 pairs of normal tissue and cancerous tissue arrays available for analysis of these specific genes.

### 2.1. Statistical Analysis

We analyzed the log_2_ RNA expression of the selected genes in normal and cancerous tissues for statistical significance using a paired *t*-test with GraphPad Prism software (version 8.0 for windows, GraphPad Inc., San Diego, California, USA).

In some cases, the data was not normally distributed based upon the D'Agostino and Pearson normality test and/or the Shapiro-Wilk normality test, whereupon comparisons between the normal and cancer tissue were made using the Wilcoxon matched-pairs signed rank test with GraphPad Prism software (version 8.0 for windows, GraphPad Inc., San Diego, California, USA). The nonnormally distributed expression of RAS-related genes in the tumor samples were *ATP6AP2* (prorenin receptor), *PREP* (prolyl endopeptidase), *LNPEP* (Ang IV receptor), and *NLN* (neurolysin) which were negatively skewed, as well as *ANPEP* (aminopeptidase N) which was positively skewed. Of note, two normal tissue gene expression distributions were also nonnormally distributed: *PREP* (prolyl endopeptidase), which was negatively skewed, and *LNPEP* (Ang IV receptor), which was positively skewed. All the nonnormally distributed genes showed kurtosis, meaning that there was an excess of values to the left or right of the average depending on whether the values were negatively or positively skewed, respectively.

Two levels of significance are reported: one which is not corrected for multiple comparison in view of the large number of comparisons that were made and one that was corrected for the multiple comparisons (Figures 2–4, S1-S2). The uncorrected significance level is reported because the likelihood of making a type II error (failure to reject a false null hypothesis) increases with the number of multiple comparisons, albeit the chances of making a type I error (failure to accept a true null hypothesis) also increases. For 17 comparisons using the Sidak's correction at a level of *p* ≤ 0.05 after correction, the significance level would need to be *p* < 0.003013 = [1 − (1 − 0.05)^1/17^]. For *p* < 0.01 after correction, the significance level would need to be *p* < 0.000591 = [1 − (1 − 0.01)^1/17^].

Tissue stages of tumor samples were based upon TNM staging as described by Tsukamoto et al. [83]. All nonredundant tumor samples (*N* = 108) were analyzed with log_2_ RNA expression of the 17 genes at different stages of cancer using a one-way ANOVA with post hoc Bonferroni comparisons. Values shown are mean ± SEM or median where the sample set did not have a normal distribution.

### 2.2. Literature Search Terms

The literature search used PubMed with the following key words: renin-angiotensin system, colorectal cancer, angiotensin metabolism, and angiotensin receptors, in combination or alone, with/without the additional search term review. In addition, derivative references were obtained from review articles found in the original literature search.

## 3. Results

### 3.1. Gene Expression in Normal versus Cancerous Colorectal Tissue

The changes in gene expression for each of the 17 RAS-related genes are described in Figures 2–4, S1-S2, and Table 1. Both the corrected and uncorrected levels of significance are shown with corrected levels indicated as *p* < 0.05 or *p* < 0.01 in Table 1. Genes of the classical RAS showing significant differences at the *p* < 0.01 level after correction are shown in Figure 2, while genes encoding nonclassical RAS-related proteins showing significant differences at the *p* < 0.01 level after correction are shown in Figure 3. Genes encoding prorenin receptor (*ATP6AP2*) and aminopeptidase A (*ENPEP*) which showed significant differences at the *p* < 0.05 level after correction are displayed in Figure 4. The remaining gene expression values which were not significant after correction for multiple comparisons are reported in Figures S1-S2.

There were significant increases in gene expression for angiotensinogen (*AGT*), aminopeptidase A (*ENPEP*), prorenin receptor (*ATP6AP2*), neprilysin (*MME*), and prolyl endopeptidase (*PREP*), while there were significant decreases in gene expression for renin (*REN*), aminopeptidase N (*ANPEP*), Mas receptor (*MAS1*), thimet oligopeptidase (*THOP*), and neurolysin (*NLN*). There were nonsignificant (after correction for multiple comparisons) trends for increases in gene expression for prolylcarboxypeptidase (*PRCP*) and the AT_2_ receptor (*AGTR2*), while there were similarly nonsignificant trends for decreases in gene expression for angiotensin-converting enzyme (*ACE*) and chymase (*CMA1*).

The relative expression of genes of the RAS and RAS-related enzymes varied considerably in tumor tissue (Table 1, Figures 2–4, and S1, S2), with the prorenin receptor having the highest expression followed by prolyl endopeptidase, ACE2, and angiotensinogen. The lowest relative expression of genes of the RAS and RAS-related enzymes was chymase, with renin and the AT_2_ receptor also showing low relative expression.

There were no systematic differences in relative gene expression of the RAS and RAS-related enzymes with the stage of the tumor. There was a marginally significant reduction in gene expression for *PRCP*, *ACE2*, and *AGT* in stage 2B relative to stage 1, but this did not approach statistical significance for a multiple comparison correction. In general, gene expression was consistent for all genes surveyed across all stages and did not show evidence for trends toward increases or decreases with increasing stage number. A representative example wherein expression of *AGT* in stage 2B was significantly lower than those in stages 1 and 4 (*p* < 0.01) by post hoc Bonferroni comparison is shown in Figure S3.

#### 3.1.1. Angiotensinogen (*AGT*)

There was a large highly significant increase of 2.413 log units in *AGT* gene expression suggesting increased production of the angiotensinogen precursor of the angiotensin peptides in colorectal tumor tissue (Table 1).

#### 3.1.2. Renin (*REN*)

There was a highly significant reduction of −0.4336 log units of *REN* gene expression, which could indicate reduced Ang I formation and buildup of angiotensinogen (Table 1).

#### 3.1.3. Prorenin Receptor (*ATP6AP2*)

There was no significant change in expression of this receptor in normal versus cancerous tissue samples.

#### 3.1.4. Chymase (*CMA1*)

There was no significant change in expression of this receptor in normal versus cancerous tissue samples.

#### 3.1.5. Neprilysin (*MME*)

There was a highly significant increase of 1.809 log units in neprilysin (CD10, CALLA) gene expression in colorectal tumor tissue (Table 1). Neprilysin metabolizes Ang I to form Ang 1-7, competing with ACE, thereby reducing the formation of Ang II. However, neprilysin metabolizes a wide variety of peptides including atrial natriuretic peptide which is the basis for the use of sacubitril, a neprilysin inhibitor in the heart failure drug, Entresto®.

#### 3.1.6. Neurolysin (*NLN*)

Neurolysin has a similar role in the RAS as neprilysin (*MME*). There was a highly significant decrease, −0.41 log units, in expression of *NLN* in CRC tissues (Table 1). In view of the increased expression of *MME* and decreased expression of *NLN*, but with higher gene expression of *NLN* (Table 1), the change in formation of Ang 1-7 from Ang I would likely be small.

#### 3.1.7. Angiotensin-Converting Enzyme (*ACE*)

There was a decrease in expression of *ACE* gene in CRC tissues that was significant only in the uncorrected comparison (Table 1). ACE is responsible for the conversion of Ang I to Ang II. A decrease of ACE expression in CRC tissues implies a reduction in the conversion of Ang I to Ang II, known for its vasoconstrictive properties.

#### 3.1.8. Angiotensin-Converting Enzyme 2 (*ACE2*)

There was an insignificant decrease in expression of *ACE2* gene in CRC tissues. ACE2 inactivates Ang II by forming Ang 1-7, the putative agonist for the Mas receptor.

#### 3.1.9. Thimet Oligopeptidase (*THOP*)

There was no significant change in expression of this enzyme in normal versus cancerous tissue samples.

#### 3.1.10. Aminopeptidase A (*ENPEP*)/Aminopeptidase N (*ANPEP*)

There was a significant increase of −0.9306 log units in *ENPEP* gene expression, suggesting an increase in aminopeptidase A-mediated conversion of Ang II to Ang III. Additionally, there was a highly significant decrease of 4.253 log units in *ANPEP* gene expression, suggesting a decrease in aminopeptidase N-mediated conversion of Ang III to Ang IV. These changes would greatly increase the amount of Ang III in tumor tissue, which could indicate that Ang III might be a better tumor promoter than Ang II.

#### 3.1.11. AT_4_ Receptor/Insulin-Related Aminopeptidase (*LNPEP*)

There was no significant change in expression of this receptor/enzyme in normal versus cancerous tissue samples.

#### 3.1.12. Prolyl Carboxypeptidase (*PRCP*)

There was an increase in *PRCP* gene expression in CRC tissue samples compared to their normal counterpart, but it was significant only in the uncorrected comparison (Table 1). PRCP also mediates inactivation of Ang II by metabolizing it to Ang 1-7.

#### 3.1.13. Prolyl Endopeptidase (*PREP*)

There was no significant change in expression of this enzyme in normal versus cancerous tissue samples.

#### 3.1.14. Type 1 AT1R Ang II Receptor (*AGTR1*) and Type 2 AT2R Ang II Receptor (*AGTR2*)

AT_1_ and AT_2_ receptor gene expression was unchanged in normal and in cancerous tissues.

#### 3.1.15. Mas (*MAS1*)

There was a highly significant reduction of 0.985 log units in *MAS1* gene expression in CRC tissue.

## 4. Discussion

Components of the modern-day RAS (Figure 1) and their role in various cancer pathways have been described recently with attention to the quantitative expression of genes in cancerous tissues and their normal tissue counterparts. Multiple studies have described a possible role of the RAS in various types of cancer, including lung cancer, breast cancer [60, 84–87], CRC, and CRC liver metastases [15, 27] (Table 2). There is considerable evidence of a relationship between polymorphisms in ACE and gastric cancer [88], lung cancer [89, 90], prostate cancer [91], and cancer in general [92].

We were particularly interested in recent studies that describe the RAS in CRC primary and metastatic tissues. The studies that focused on RAS components in CRC generally found a consistent correlation between RAS-related gene expression in CRC tissues with ACE, MasR, AT1R, and AT2R expression being altered in CRC primary and metastatic tissues [15, 27]. Protumoral associations of the RAS proteins may be related to gliomas as well [26].

We observed statistically significant alterations in gene expression of many, but not all, RAS-related components in CRC specimens. The significant increase in angiotensinogen gene expression in the CRC (Figure 2) is suggestive of an increased supply of the precursor protein of the RAS leading to a general increase in activity of the system. However, the genes encoding the enzymes that process angiotensinogen, renin and ACE, to form Ang II, are decreased in the CRC samples compared to normal tissue, so it is not possible to speculate whether Ang II formation is increased or decreased. It is possible that there may be non-RAS-mediated effects of the increased angiotensinogen in the CRC tissues. Both angiotensinogen and des-Ang I angiotensinogen promote weight gain and liver steatosis in mice that are independent of the RAS [93]. Interestingly, angiotensinogen-deficient mice exhibited an increase in vascular endothelial growth factor A (VEGF-1) which may imply that overexpression of angiotensinogen could have an antiangiogenic effect [93].

Medications that target ACE and angiotensin receptors, such as angiotensin-converting enzyme inhibitors (ACEIs) and angiotensin receptor blockers (ARBs), respectively, are widely used as antihypertensive agents and therapies for patients with heart failure and diabetic complications [94]. Newer therapeutic agents have emerged that inhibit other components of the RAS, such as neprilysin, although neprilysin has a variety of peptide substrates other than angiotensins. Accordingly, if the proteins encoded by the RAS genes play a significant role in CRC pathophysiology, then, already existing therapies could potentially treat CRC.

The expression of genes encoding the major receptors for angiotensin peptides, AT1R and AT2R, was not different between tumor and normal tissue samples (Figures S1 and S2) which might argue against a major pathophysiological involvement of the classical RAS in CRC despite the mitogenic potential of AT1R signaling [95]. Increased AGTR1 gene expression is associated with breast cancer [85], and ARBs inhibit mammary tumor formation in mice [86, 87]. Similar, to the observation of no increase in AT1R gene expression in this study, ARB usage did not have a protective effect against CRC in a retrospective study of a large Spanish population [96].

Receptor stimulation also depends upon agonist availability, which is subject to regulation by metabolic activity. Relevant to angiotensins, there is a substantial increase in aminopeptidase A gene expression. Aminopeptidase A is the enzyme that metabolizes Ang II to form Ang III, suggesting a reduction in the degree to which Ang II would be able to stimulate AT_1_ and AT_2_ receptors. With respect to Ang III, there was a profound decrease in aminopeptidase N gene expression in the tumor tissue. Aminopeptidase N is the major inactivating enzyme for Ang III, so a reduction in its expression coupled with an increase in aminopeptidase A expression would cause a substantial accumulation of Ang III-mediated activation of AT_1_ and AT_2_ receptors. Also, reduced activity of aminopeptidase N would decrease Ang IV formation, reducing stimulation of the AT_4_ receptor. However, there was also no significant change in AT_4_ receptor gene expression, making it unlikely that there was a significant alteration in AT_4_ receptor signaling in the CRC tumor tissue. Of note, blockade of aminopeptidase A in the brain is reported to decrease stimulation of AT_1_ receptors, implying that Ang III is a more efficacious agonist than Ang II on brain AT_1_ receptors [65, 97]. Ang III was reported to be more potent than Ang II in the rat brain [98], although Ang III is generally considered to be near equipotent with Ang II as an AT_1_ receptor agonist [63]. Also, it was recently shown that Ang II and Ang III signal at AT_1_ receptors with similar potency for G protein and beta-arrestin-mediated signaling pathway profiles [99]. Thus, the evidence for involvement of altered AT_1_ or AT_2_ receptor activation in tumor tissue in this cohort is mixed.

Studies have previously demonstrated that established therapies, particularly ACE inhibitors and ARBs, have a role in reducing the risk of cancers, improving cancer survival outcomes, delaying progression of invasive cancers, and decreasing the quantity of tumor metastasis [15, 17, 18, 27, 86, 100, 101]. These studies have looked at RAS-related medications in CRC and CRC metastasis, adenomatous polyps, breast, prostate, renal, and small cell cancers (Table 3).

ACEIs or ARBs, when used in conjunction with COX-2-selective inhibitors, resulted in the downregulation of tumor growth in CRC patients [17]. Additionally, they found that when used for 3 or more years, ACEIs or ARBs each resulted in a decreased risk of CRC, while CCBs used for 3 or more years resulted in no change in risk for CRC. ACEI use was found to reduce adenomatous polyps (APs) in a dose-related manner, thereby decreasing the risk of CRC via the downregulation of these CRC precursors [18].

ACEIs were shown to improve survival outcomes in breast, prostate, renal, and small cell cancers, while losartan, an ARB, slowed the invasiveness of breast cancer tumors [86]. The ACEI captopril reduced the volume of liver metastases in a mouse model of CRC [15]. However, when ACEIs and ARBs are used together, [101] there is a paradoxical increased risk of developing cancer. Fortunately, this combination of medications is rarely seen in clinical practice, because it causes more adverse drug reactions than treatment with an ACE inhibitor or ARB alone, with no improvements in key outcomes [102].

The gene expression data predicts significant alterations in RAS components in this CRC population. While we did not observe any changes in gene expression for AT_1_ and AT_2_ receptors, there were alterations in the generation and metabolism of angiotensin peptides in CRC tumor tissues that could affect AT_1_ and AT_2_ receptor signaling. It remains to be seen whether there is a genetic or environmental determinant of the tumor tissue gene expression that translates to a more robust response to therapies that block the RAS and if there is a dose-dependent mechanism that would provide patients with an optimum response to therapy. In addition, patient compliance and duration of therapy remain as possible confounders to individual patient responses. Our study further demonstrates that the RAS potentially plays a role in CRC and that the use of well-studied RAS-directed therapies, such as ACEIs, ARBs, and renin inhibitors, may be of benefit for adjunctive treatment of CRC. It is worth mentioning that many trials have been run to determine if RAS blockers can cause cancer and the evidence is overwhelmingly against any relation between RAS blockers and increased risk of cancer in general [103–106].

ACE gene expression was marginally significantly reduced in the CRC tumor samples, i.e., it was significant on its own, but not with the multiple comparison correction. This observation is in contrast to a previous study which showed an increase in ACE mRNA expression in CRC [107]. The involvement of ACE activity in tumors may be tumor specific or limited to specific ethnic groups. Having the DD (deletion) genotype of the ACE gene confers increased ACE activity and is associated with increased lymph node metastasis of CRC in a cohort of Chinese patients [16]. A meta-analysis of studies of the association of the DD and II ACE genotypes, a variety of cancers, suggested that the II ACE genotype was weakly associated with reduced risk of some cancers [108]. There is considerable variance in the reported effects of ACE inhibitors on cancer risk with some studies of CRC showing a chemoprotective effect [18, 109]. Beneficial effects of ACE inhibitors on CRC were found to be greatest in men under 65 years of age [96].

There was a substantial increase in neprilysin (CD10, CALLA) gene expression in colorectal tumor tissue. Neprilysin metabolizes Ang I to form Ang 1-7, competing with ACE, thereby reducing formation of Ang II. However, neprilysin metabolizes other peptides, which might affect tumorigenesis. Of note, neprilysin is a marker for several cancers, including leukemias [110], and is also inhibited by sacubitril, a component of the heart failure drug, Entresto® (sacubitril/valsartan). It will be of interest to determine if sacubitril or other neprilysin inhibitors affect CRC incidence. There was a substantial reduction in *MAS1* gene expression. *MAS1* encodes the Mas receptor for angiotensin 1-7 [59], which is reported to have antiproliferative properties [28]. A reduction in Mas receptor expression may facilitate unregulated proliferation of CRC cells [111]. There was also a large increase in *AGT* gene expression suggesting increased production of the angiotensinogen precursor of the angiotensin peptides in colorectal tumor tissue. However, the reduction in *REN* could indicate reduced Ang I formation and a build-up of angiotensinogen which might explain the protumor effect of angiotensinogen on CRC metastasis to the liver [112].

Finally, we examined RAS-related gene expression as a function of different stages of CRC. Although in Figure S3, expression of *AGT* in stage 2B was significantly lower than those in stages 1 and 4, gene expression was generally consistent for all genes surveyed across all stages and did not show evidence for trends toward increases or decreases with increasing stage number. This could indicate that the changes in RAS-related components are associated with tumorigenesis rather than progression of CRC. Our current analysis examines the differential genetic expression of a population of CRC patients in Japan. We plan to pursue the evaluation of the expression of these genes in surgically resected samples from locally sourced tissues to determine if the findings will translate across population demographics.

### 4.1. Limitations of Study

This Japanese population may not generalize to other ethnic groups. Alterations in gene expression do not always translate into significant alterations in protein expression and function. This study did not assess *MRGPRD* expression, which encodes Mas-related G protein receptor family member D (MrgD), one of the newer receptor components of the RAS.

## 5. Conclusion

This analysis is consistent with the involvement of both the ACE/Ang II/AT1R and ACE2/Ang 1-7/Mas axes of the RAS in CRC. However, the pathological significance of the changes in RAS-related gene expression requires continued assessment of the effects of drugs that inhibit or enhance the activities of these RAS-related components on the incidence and the survivability of CRC.

## Figures and Tables

**Table 1:** Changes in gene expression for components of the RAS and related proteins in CRC tissue samples.

Gene	Protein	Non-tumor tissue mean ± SE (log_2_)	Tumor tissue mean ± SE (log_2_)	Significance level^∗^ (uncorrected for multiple comparisons)	Significance level (corrected for multiple comparisons)^∗∗^
*AGT*	Angiotensinogen	6.93 ± 0.14	9.35 ± 0.15	*p* < 0.0001	*p* < 0.01
*REN*	Renin	5.42 ± 0.06	4.99 ± 0.07	*p* < 0.0001	*p* < 0.01
*ACE*	Angiotensin-converting enzyme	5.63 ± 0.06	5.36 ± 0.07	*p* = 0.0065	NS
*AGTR1*	AT_1_ Ang II receptor subtype	6.05 ± 0.05	5.98 ± 0.04	*p* = 0.23	NS
*AGTR2*	AT_2_ Ang II receptor subtype	4.84 ± 0.02	4.9 ± 0.02	*p* = 0.011	NS
*ATP6AP2*	Prorenin receptor	10.6 ± 0.04	10.9 ± 0.13	*p* = 0.0031	p =0.05
*CMA1*	Chymase	4.66 ± 0.04	4.55 ± 0.04	*p* = 0.043	NS
*LNPEP*	Ang IV receptor, insulin-regulated aminopeptidase	8.28 ± 0.09	8.47 ± 0.01	*p* = 0.055	NS
*ENPEP*	Aminopeptidase A	6.71 ± 0.14	7.7 ± 0.14	*p* = 0.0011	*p* < 0.05
*ANPEP*	Aminopeptidase N	9.85 ± 0.36	5.6 ± 0.21	*p* < 0.0001	*p* < 0.01
*ACE2*	Angiotensin-converting enzyme-2	9.86 ± 0.11	9.42 ± 0.37	*p* = 0.283	NS
*MME*	Neprilysin	5.7 ± 0.11	7.510.31	*p* < 0.0001	*p* < 0.01
*PRCP*	Prolyl carboxypeptidase	7.31 ± 0.06	7.7 ± 0.10	*p* = 0.0073	NS
*PREP*	Prolyl endopeptidase	8.61 ± 0.13	9.47 ± 0.11	*p* < 0.0001	*p* < 0.01
*MAS1*	Mas, Ang 1-7 receptor	7.05 ± 0.09	6.07 ± 0.05	*p* < 0.0001	*p* < 0.01
*NLN*	Neurolysin	9.36 ± 0.06	8.95 ± 0.07	*p* = 0.0004	*p* < 0.01
*THOP*	Thimet oligopeptidase	8.2 ± 0.11	7.28 ± 0.12	*p* < 0.0001	*p* < 0.01

^∗^Paired *t*-test; ^∗∗^Sidak's correction: (1 − (1 − 0.05)^1/17^) or (1 − (1 − 0.01)^1/17^) or (1 − (1 − 0.001)^1/17^); NS is nonsignificant.

**Table 2:** Change of the RAS gene expression in various cancers.

Cancer type	RAS gene	RAS protein	Change	Citation
Invasive duct cell breast cancer	*MMP*	Neprilysin	Downregulated	Stephen et al. [113]

CRC liver metastases	*AGTR1*	AT1R	Upregulated	Neo et al. [15], Zhou et al. [27]
	*AGTR2*	AT2R	Upregulated	Neo et al. [15]
	*AGT*	Angiotensinogen	No change	Neo et al. [15]
	*ACE*	Angiotensin-converting enzyme	Upregulated	Neo et al. [15]
	*MAS1*	Mas receptor	Upregulated	Neo et al. [15]

CRC	*AGTR1*	AT1R	Upregulated, protumoral, and dose dependent on Ang II concentration	Zhou et al. [27]
	*AGTR2*	AT2R	Biphasic, Ang II dose dependent (low = protumoral, high = antitumoral)	Zhou et al. [27]
	*AGTR2*	AT2R	Protumor at low (Ang II), antitumoral effects at high (Ang II)	Zhou et al. [27]

Breast cancer	*AGTR2*	AT2R	Upregulated	Zhou et al. [27]

Pancreatic cancer	*AGTR2 via ATII*	AT2R via ATII	Upregulated	Zhou et al. [27]

Chemically induced lung cancer	*AGTR2*	AT2R	Upregulated	Zhou et al. [27]

CRC, lymph node metastases	*ACE*	Angiotensin-converting enzyme	Allele dependent	Zheng et al. [16]

**Table 3:** Use of RAS inhibitors and other medications in various cancers.

Medication	Cancer type	Effect of medication on cancer type	Citation
ACEI/ARBs + COX − 2 inhibitors	CRC	Downregulated tumor growth	Makar et al. [17]
ACEI/ARB ≥ 3 years	CRC	Decreased RISK of CRC	Makar et al. [17]
CCB (high dose) ≥ 3 years	CRC	No change	Makar et al. [17]
Statins, ACEI, CCBs, diuretics	CRC	No change in risk of CRC	Boudreau et al. [100]
ACEI (dose related)	Adenomatous polyps	Decreased risk of CRC via downregulation of adenomatous polyps	Kedika et al. [18]
ACEI and ARB combination	Cancer (nonspecific)	Increased risk of cancer	Bangalore et al. [101]
ACEI	Breast, prostate, renal, and small cell cancer	Improved survival outcomes	Coulson et al. [86]
Losartan (ARB)	Breast Cancer	Delays occurrence and progression of invasive breast cancer	Coulson et al. [86]
Captopril (ACEI)	CRC liver metastases	Decreased tumor metastases	Neo et al. [15]

## Data Availability

Raw data for this manuscript can be made available upon request for scientific review by qualified parties.

## Abbreviations

ACE: Angiotensin-converting enzyme
*ACE*: Angiotensin-converting enzyme gene
ACE2: Angiotensin-converting enzyme-2
*ACE2*: Angiotensin-converting enzyme-2 gene
ACEI: Angiotensin-converting enzyme inhibitor
*AGT*: Angiotensinogen gene
*AGTR1*: AT_1_ receptor gene
*AGTR2*: AT_2_ receptor gene
ANG: Angiotensin
Ang I: Angiotensin I
Ang II: Angiotensin II
Ang III: Angiotensin III
Ang IV: Angiotensin IV
*ANPEP*: Aminopeptidase N gene
AP: Adenomatous polyp
ARB: Angiotensin receptor blockers
AT1R: AT_1_ Ang II receptor subtype
AT2R: AT_2_ Ang II receptor subtype
*ATP6AP2*: Prorenin receptor gene
CCB: Calcium channel blockers
*CMA1*: Chymase gene
COX: Cyclooxygenase
CRC: Colorectal cancer
*ENPEP*: Aminopeptidase A gene
FAP: Familial adenomatous polyposis
IRAP: Insulin-regulated aminopeptidase
*LNPEP*: Insulin-regulated aminopeptidase (Ang IV receptor) gene
*MAS1*: Mas receptor gene
*MME*: Neprilysin gene
*NLN*: Neurolysin gene
PRCP: Prolylcarboxypeptidase
*PRCP*: Prolyl carboxypeptidase gene
*PREP*: Prolyl endopeptidase gene
RAS: Renin-angiotensin system
*REN*: Renin gene
SEM: Standard error of the mean
*THOP*: Thimet oligopeptidase gene.

## Supplementary Materials

Supplementary Materials Figure S1: RAS components and related enzymes that showed only uncorrected significant differences in gene expression between normal and tumor tissues. From top to bottom, the panels describe angiotensin-converting enzyme gene (*ACE*) expression, chymase gene (*CMA1*) expression, AT_2_ receptor gene (*AGTR2*) expression, and prolyl carboxypeptidase gene (*PRCP*) expression; left panels show pairing of samples with connecting lines. Right panels show mean, SEM, and individual data points. All of these comparisons were significant at the *p* < 0.05 level before correction for multiple comparisons but were not significant after correction for multiple comparisons. Figure S2: RAS components that did not show any significant differences in *gene* expression between normal and tumor tissues. Top panels describe AT_1_ receptor gene (*AGTR1*) expression, middle panels describe AT_4_ receptor gene (*LNPEP*) expression, and lower panels describe angiotensin-converting enzyme-2 gene (*ACE2*) expression. Left panels show pairing of samples with connecting lines. Right panels show mean, SEM, and individual data points. Figure S3: relative expression of angiotensinogen gene (*AGT)*, with different stages of CRC at time of biopsy. One-way ANOVA revealed a significant (*F*_6,105_ = 3.117, *p* = 0.0075) effect of the stage on gene expression. ^∗∗^Post hoc Bonferroni test indicated that *AGT* gene expression during stage 2B was significantly less (*p* < 0.01) than those during stage 1 or stage 4.
