# Supplementary material for: Alterations in Gene Expression of Renin-Angiotensin System Components and Related Proteins in Colorectal Cancer
Source: J Renin Angiotensin Aldosterone Syst. 2021 Jul 5;2021:9987115. doi: 10.1155/2021/9987115 (PMC8277508; doi:10.1155/2021/9987115)
Supplement: Supplementary Materials — Figure S1: RAS components and related enzymes that showed only uncorrected significant differences in gene expression between normal and tumor tissues. From top to bottom, the panels describe angiotensin-converting enzyme gene (ACE) expression, chymase gene (CMA1) expression, AT2 receptor gene (AGTR2) expression, and prolyl carboxypeptidase gene (PRCP) expression; left panels show pairing of samples with connecting lines. Right panels show mean, SEM, and individual data points. All of these comparisons were significant at the p < 0.05 level before correction for multiple comparisons but were not significant after correction for multiple comparisons. Figure S2: RAS components that did not show any significant differences in gene expression between normal and tumor tissues. Top panels describe AT1 receptor gene (AGTR1) expression, middle panels describe AT4 receptor gene (LNPEP) expression, and lower panels describe angiotensin-converting enzyme-2 gene (ACE2) expression. Left panels show pairing of samples with connecting lines. Right panels show mean, SEM, and individual data points. Figure S3: relative expression of angiotensinogen gene (AGT), with different stages of CRC at time of biopsy. One-way ANOVA revealed a significant (F6,105 = 3.117, p = 0.0075) effect of the stage on gene expression. ∗∗Post hoc Bonferroni test indicated that AGT gene expression during stage 2B was significantly less (p < 0.01) than those during stage 1 or stage 4. [file 9987115.f1.zip › For Production- correct submission date.docx]

*This article is a transfer so the submission date needs updating. The correct submission date is* Feb 2, 2021
